# LP1 from *Lentinula edodes* C_91-3_ Induces Autophagy, Apoptosis and Reduces Metastasis in Human Gastric Cancer Cell Line SGC-7901

**DOI:** 10.3390/ijms19102986

**Published:** 2018-09-30

**Authors:** Samana Batool, Thomson Patrick Joseph, Mushraf Hussain, Miza S. Vuai, Kavish. H. Khinsar, Syed Riaz Ud Din, Arshad Ahmed Padhiar, Mintao Zhong, Anhong Ning, Wei Zhang, Jing Cao, Min Huang

**Affiliations:** 1 Department of Microbiology, College of Basic Medical Sciences, Dalian Medical University, Dalian 116044, China; samana.batool12@yahoo.com (S.B.); microbiologist02@gmail.com (T.P.J.); silima.miza@outlook.com (M.S.V.); kavishhasnain@ymail.com (K.H.K.); riazuddindmu@outlook.com (S.R.U.D.); arshad.padhiar@outlook.com (A.A.P.); dyzhongmt@163.com (M.Z.); yynah-64@163.com (A.N.); zhangw@dlmedu.edu.cn (W.Z.); caojingaizhe@163.com (J.C.); 2 State Key Laboratory of Fine Chemicals, School of Chemical Engineering, Dalian University of Technology, E-208 West Campus, Dalian 116024, China; mushraf_chemist@yahoo.com

**Keywords:** *Lentinula edodes*, Latcripin 1, gastric cancer, autophagy, apoptosis, metastasis

## Abstract

Present study aimed to elucidate the anticancer effect and the possible molecular mechanism underlying the action of Latcripin 1 (LP1), from the mushroom *Lentinula edodes* strain C_91-3_ against gastric cancer cell lines SGC-7901 and BGC-823. Cell viability was measured by Cell Counting Kit-8 (CCK-8); morphological changes were observed by phase contrast microscope; autophagy was determined by transmission electron microscope and fluorescence microscope. Apoptosis and cell cycle were assessed by flow cytometer; wound-healing, transwell migration and invasion assays were performed to investigate the effect of LP1 on gastric cancer cell’s migration and invasion. Herein, we found that LP1 resulted in the induction of autophagy by the formation of autophagosomes and conversion of light chain 3 (LC3I into LC3II. LP1 up-regulated the expression level of autophagy-related gene (Atg7, Atg5, Atg12, Atg14) and Beclin1; increased and decreased the expression level of pro-apoptotic (Bax) and anti-apoptotic (Bcl-2) proteins respectively, along with the activation of Caspase-3. At lower-doses, LP1 have shown to arrest cells in the S phase of the cell cycle and decreased the expression level of matrix metalloproteinase MMP-2 and MMP-9. In addition, it has also been shown to regulate the phosphorylation of one of the most hampered gastric cancer pathway, that is, protein kinase B/mammalian target of rapamycin (Akt/mTOR) channel and resulted in cell death. These findings suggested LP1 as a potential natural anti-cancer agent, for exploring the gastric cancer therapies and as a contender for further in vitro and in vivo investigations.

## 1. Introduction

Gastric cancer is one of the world’s leading causes of cancer-associated death, with a minimum survival rate of five years after diagnosis [1]. American cancer society revealed the expectation of approximately 26,240 new cases and about 10,800 mortalities in the United States alone by the end of year 2018 [2]. East Asian countries such as China, Japan and Korea are at higher risk of gastric cancer as compared to rest of the world [3]. Gastric cancer mostly develops in many stages with the initiation of malignant cells in the inner lining of the stomach which further extends from second to the third layer and infects the lymph nodes [4]. Gastric cancer may spread from the stomach to other parts of the body including but not limited to the lungs, liver and peritoneum layer [5]. In 60% of the cases, *Helicobacter pylori* has been reported to be the main culprit, while in rest of the cases several lifestyle (smoking, dietary habits, obesity) associated and genetic factors are involved [6]. Although it takes several years for the development of stomach cancer however, initial symptoms including anorexia, dyspepsia, weight loss and abdominal discomfort are mostly ignored by the patients [7]. Early diagnosis of gastric cancer is very crucial, as in the advanced stages treatment is difficult because of the metastasis which leads the degradation of extracellular matrix, epithelial-to-mesenchymal transition and abnormalities in programmed cell death [8]. Although a number of novel anticancer agents and strategies have improved the treatment regime against gastric cancer but most of them possess several side effects. Surgery is often suggested to the patient at an early stage but in later stages recurrence is a common problem [9]. Chemotherapy is considered as an alternative method for the treatment of gastric cancer however, in clinical applications, drug resistance and toxicity are the main hurdles [10].

A natural treatment for a disease is always the best choice due to its minimum side effects, easy availability and low cost. Among natural products, mushrooms have a long history to be used as a source of food and medicine [11]. One of the most cultivated mushrooms is *Lentinula edodes*, which is associated with a number of medicinal properties ranging from anti-microbial to anti-metastatic and anti-cancer to immunomodulation [12,13,14]. The clinical trials on gastric cancer patients proved *Lentinula edodes* as a worthy source to minimize the severe side effects of chemotherapy [15]. In this regard, our research group have expressed and isolated a number of proteins from *Lentinula edodes* C_91-3_ and investigated their effects on different kinds of cancer cell lines. For instance, LP3 exhibited a good efficacy against lung cancer cell line A549 by arresting the S phase of cell cycle and inducing apoptosis [16]. Anticancer role of LP13 was revealed by cell cycle arrest at G_1_ phase and apoptosis by NF-κB Signaling pathway in A549 cell line [17]. Lp16-PSP inhibited anchorage-independent growth; p21^WAF1/CIP1^ mediated cell cycle arrest at the G_1_ phase and apoptosis by the inhibition of NF-κB in leukemia cell line HL-60 [18].

As far as the anticancer potential of LP1 is a concern, its role in the induction of apoptosis [19] and autophagy [20] has been reported previously in lung cancer cell line A549 however, its effect on other cancer cell lines has not been explored yet. Hence, in order to investigate the anticancer potential of LP1 against other cancer types, a panel of cancer cell lines was subjected to LP1 (as expressed previously) [20] and we identified gastric cancer cell lines (SGC-7901 and BGC-823) as the most sensitive cell lines. Thus, we preceded our detailed investigation with SGC-7901 and also performed some key experiments (for autophagy and apoptosis) on BGC-823 in order to explore the cell type dependency of LP1 protein.

## 2. Results

### 2.1. LP1 Inhibits Cell Viability and Cell Proliferation

**Figure 1:**
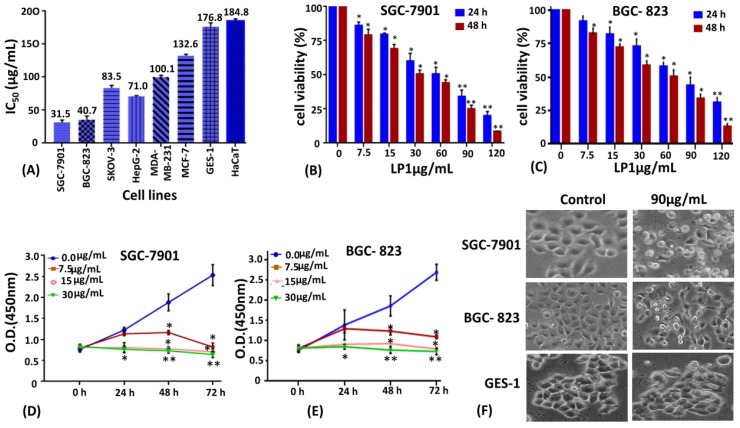
Effect of LP1 on the viability and proliferation of SGC-7901 and BGC-823 cells. (**A**) IC_50_ values of LP1 against a panel of cancerous and non-cancerous cell lines were evaluated and results are expressed as means ± SD (Standard Deviation) of three independent experiments at 48 h. (**B**) SGC-7901 cells and (**C**) BGC-823 cells treated with different concentration of LP1 for 24 and 48 h, to determine cell viability by CCK-8. Results are expressed as a percentage of corresponding control and means ± SD of three independent experiments; (* *p* < 0.05, ** *p* < 0.01). (**D**,**E**) Cell proliferation assay performed by CCK-8, with a low dose of LP1 at different time intervals for SGC-7901 and BGC-823 cells. Results are presented as means ± SD of three independent experiments; (* *p* < 0.05, ** *p* < 0.01). (**F**) Morphological changes in SGC-7901, BGC-823 and GES-1 cell lines were observed under a phase contrast microscope after 48 h treatment with a mentioned concentration of LP1 at 40× magnification.

The CCK-8 kit assay was performed to assess the effect of LP1 on cancer cell lines (SGC-7901, BGC-823, SKOV-3, HepG-2, MDA-MB-231, MCF-7) and normal cell lines (GES-1 and HaCaT) with varying concentrations (0 to 120 µg/mL) of LP1 for 48 h; IC_50_ was calculated for all cell lines (Figure 1A). IC_50_ of LP1 was 31.5 and 40.7 µg/mL for SGC-7901 and BGC-823 cells respectively, which is lower as compared to other cancer cell lines. Similarly, LP1 inhibit cellular proliferation (50%) of normal cell lines GES-1 and HaCaT at relatively higher doses (176.8 and 184.8 µg/mL respectively). CCK-8 was also used to determine cell viability for SGC-7901 and BGC-823 cells at two different time intervals (Figure 1B,C). Cell proliferation assay was conducted to assess the rate of cell growth inhibition with low doses of LP1 (0, 7.5, 15 and 30 µg/mL) at different time intervals (0, 24, 48 and 72 h). The results indicated significant growth inhibition by LP1 in a dose and time-dependent-manner (Figure 1D,E). The morphological changes in SGC-7901 and BGC-823 cells were observed under a phase contrast microscope and presented (Figure 1F). Images of phase contrast microscope showed that the SGC-7901 cells were ruptured and deformed in shape as compared to the untreated cells which possessed a polygonal shape. Similarly, LP1 treated, BGC-823 cells were also ruptured and deformed in shape as compared to the control group. However, minor morphological changes were observed in GES-1 normal gastric cell line when treated with LP1 (Figure 1F). These results suggested that LP1 had a promising inhibitory effect on the cell viability and proliferation in SGC-7901 and BGC-823 cells as compared to control in a dose and time-dependent-manner.

### 2.2. LP1 Arrested S Phase of the Cell Cycle in SGC-7901 Cells

**Figure 2:**
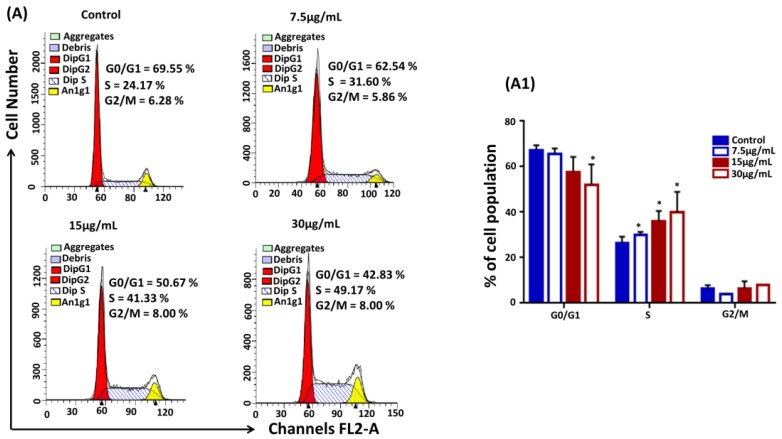
LP1 arrested S phase of the cell cycle. (**A**) SGC-7901 cells treated with (0, 7.5, 15, 30 µg/mL) of LP1 for 48 h to analyze DNA content by using flow cytometer. Representative images indicating the distribution and percentage of cells in G0/G1, S and G2/M phase. (**A1**) The graph represents the distribution and percentage of cells within different phases of the cell cycle. Results are expressed as means ± SD of three independent experiments and (* *p* < 0.05).

To investigate the effect of LP1 on the cell cycle distribution, SGC-7901 cells were treated with mentioned concentrations of LP1 for 48 h. Cells were stained with Propidium iodide (PI) and prepared for the cell cycle analysis by FACS-Calibur Cytometer. LP1 treated cells were arrested mostly in S phase of the cell cycle. The percentage of arrested cells’ population considerably increased from 31% to 49% in treated groups whereas in control group it was 24%; while the percentage of cell population decreased in G0/G1 phase (Figure 2A and Figure 2(A1)). These results suggested that LP1 had a significant impact to interrupt the cell cycle progression of SGC-7901 cells in a concentration dependent-manner.

### 2.3. LP1 Induced Autophagy in SGC-7901 and BGC-823 Cell Lines

**Figure 3:**
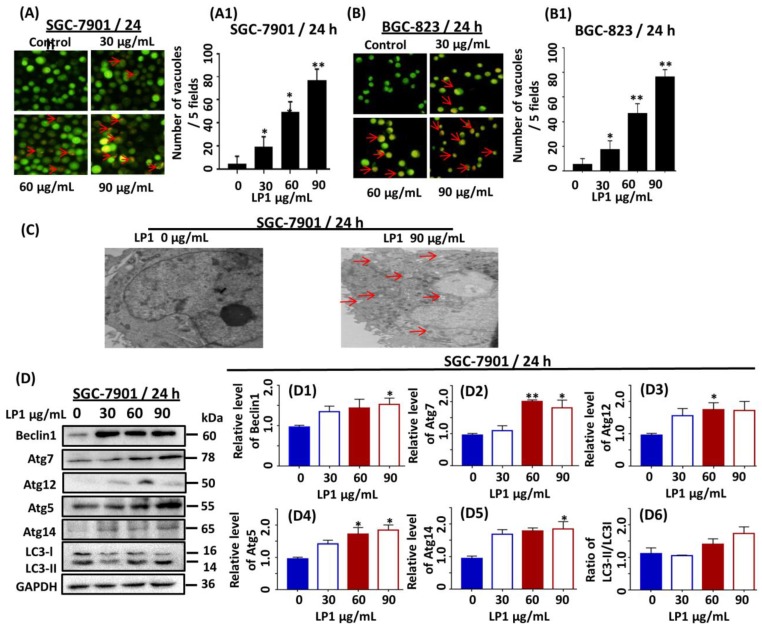
Investigating the role of LP1 in the induction of autophagy by acridine orange staining (in SGC-7901 & BGC-823 cells), electron microscopy and western blotting in SGC-7901 cells. (**A**,**B**) Fluorescence microscope was used to take images of acidic vesicular organelles (AVOs), scale bar 100 µm was used for these images, SGC-7901/BGC-823 cells were treated with various concentration (0, 30, 60, 90 µg/mL) of LP1 for 24 h; before staining with acridine orange. (**A1**,**B1**) Numbers of vacuoles per cell were quantitated in five different fields. Results are expressed as means ± SD and (* *p* < 0.05, ** *p* < 0.01) of three independent experiments. (**C**) SGC-7901 cells were treated with and without LP1 for 24 h and electron microscopy was performed at 8000×; where red arrow indicating the autophagosomes. (**D**) The expression level of Beclin1, Atg7, Atg5, Atg12 Atg14 and LC3 in SGC-7901 cells treated with various concentration (0, 30, 60, 90 µg/mL) of LP1 for 24 h, analyzed by western blot assay. GAPDH was used as an internal control. (**D1**–**D6**) Western blots were quantified by densitometry, results are expressed as mean ± SD and (* *p <* 0.05, ** *p* < 0.01) of three independent experiments.

SGC-7901 and BGC-823 cells were stained with acridine orange dye and examined under a fluorescence microscope. Acridine orange is an organic basic dye, capable of penetrating through the cell membrane which emits green light under a neutral environment. It facilitates to study the autophagy vacuolization within the cells by emitting orange light upon protonation in acidic parts of the cells such as lysosome. We observed that LP1 treated SGC-7901 and BGC-823 cells exhibited orange fluorescence (Figure 3A,B) which is a characteristic feature of autophagy. Moreover, we statistically analyzed the numbers of vacuoles and found that they are significantly increased in treated groups as compared to the untreated group in a dose-dependent fashion (Figure 3(A1,B1)). For further confirmation, electron microscopy was performed to observe the ultrastructure of SGC-7901 cells and results indicated the presence of a large number of autophagic vesicles in treated cells as compared to the control cells (Figure 3C). LC3 is considered as a hallmark in the determination of autophagy; during autophagy it converts from LC3I to LC3II. To assess the conversion of LC3 from cytoplasmic form to lipid form, the expression level of LC3 was investigated by means of western blotting (Figure 3D). The results indicated that the conversion ratio of LC3II over LC3I was 1.2 and 1.5-fold higher in the treated group (60 µg/mL and 90 µg/mL) respectively as compared to control group in a dose-dependent manner (Figure 3(D6)). It is reported that Beclin1 forms complex with the PI3KCIII which is involved in the initiation of autophagic vacuoles. The Beclin1 is a tumor suppressor gene responsible for the regulation of autophagy, cell mortality and defends the immune system [21]. In yeast, PI3KCIII forms two kinds of complexes with Atg14 and vacuolar protein sorting (Vps34) which induces autophagy and endocytosis respectively [22]. Atg14 is a mammalian orthologue of Atg14L that is a component of the PI3KCIII**-**Beclin1 complex and essential for the development of autophagosomes [23,24]. We measure the expression level of Beclin1 and Atg14 by western blotting. Our results indicated 1.3, 1.4 and 1.6-fold increase in the expression level of Beclin1 (Figure 3(D1); * *p* < 0.05) and 1.7, 1.8 and 1.9-fold increase in the expression level of Atg14 (Figure 3(D5); * *p* < 0.05), when compared with the control cells in a dose-dependent manner. 

Ubiquitin-like proteins such as Atg7 has an important role in the formation of a covalent conjugated bond between Atg5 and Atg12 which further function in autophagic membranes elongation [23,24]. These membranes accumulate all around the spoiled old proteins, cytoplasmic parts and intracellular organelles and are called as autophagosomes that are finally degraded as lysosomes [25]. To understand this phenomenon, we conducted western blotting experiments and results indicated 1.1, 2.1 and 1.8-fold increase in the expression level of Atg7 (Figure 3(D2)); * *p* < 0.05, ** *p* < 0.01); 1.1, 1.7 and 1.9-fold increases in the expression level of Atg5 (Figure 3(D3)); * *p* < 0.05) and 1.6, 1.8 and 1.7-fold increase in the expression level of Atg12 (Figure 3(D4)); * *p* < 0.05) in LP1 treated groups as compared to control group.

### 2.4. LP1 Inhibited Migration, Invasion and Wound Healing in SGC-7901 Cells

**Figure 4:**
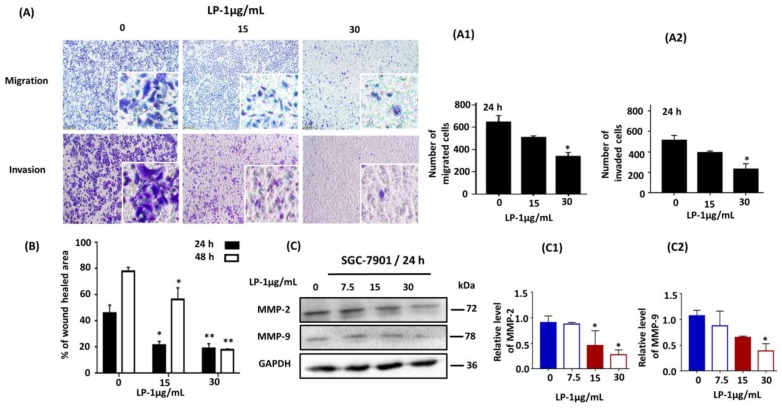
LP1 suppresses migration, invasion and wound healing in SGC-7901 cells. (**A**) Cell migration and invasion assays were performed with (0, 15, 30 µg/mL) of LP1 for 24 h. Images were captured by phase contrast microscope, scale bar 200 µm. (**A1**) A number of migrated cells quantified by using ImageJ software. (**A2**) A number of invaded cells quantified by ImageJ software. Cells were counted by three investigators in a double-blind manner. Results are expressed as means ± SD (* *p* < 0.05) of three independent experiments. (**B**) Wound healing assay was performed with 0, 15 and 30 µg/mL concentrations of LP1 in SGC-7901 cells for 24 and 48 h. Unhealed area was measured by ImageJ software in arbitrary units; results are expressed as means ± SD (* *p* < 0.05, ** *p* < 0.01); (**C**) Expression level of MMP-2 and MMP-9 in SGC-7901 cells, treated with various concentrations (0, 7.5, 13, 30 µg/mL) of LP1 for 24 h were analyzed by western blot assay, GAPDH was used as an internal control (**C1**,**C2**). Western blots were quantified by densitometry and results are expressed as mean ± SD (* *p* < 0.05) of three independent experiments.

We investigated the inhibitory effect of LP1 on the invading and migrating ability of SGC-7901 cells by cell invasion and migration assays. The transwell chambers were used with and without Matrigel matrix for cell invasion and migration assays respectively. Cells were treated with different concentrations of LP1 (0, 15 and 30 µg/mL). Cells were allowed to invade/migrate by chemotaxis towards an FBS gradient (10% FBS in the lower chamber) for 24 h (Figure 4A). The invaded/migrated cells were monitored by a phase contrast microscope after 24 h. The numbers of invaded/migrated cells were counted and found to be significantly less in LP1 treated groups as compared to control group in a concentration dependent-manner as shown in Figure 4(A1,A2). Further, we explored the effect of LP1 on wound area closure; by wound healing assay, carried out in LP1 treated (15 and 30 µg/mL) and untreated cells at 0, 24 and 48 h. The percentages of wound healed area were calculated and represented in the graph. These graphs indicated that LP1 treated cells had less capability to heal the scratch area in a dose and time dependent-manner as compared to control group (Figure 4B; * *p* < 0.05, ** *p* < 0.01).

Next, we assessed the expression level of MMP-2 and MMP-9 by conducting western blotting assay and results showed that LP1 treatment had significantly decreased the expression level of these proteins (Figure 4C). Exposure of cells to LP1 for 24 h, reduced the expression level of MMP-2 (96.6%, 49.8% and 31% for 7.5, 15 and 30 µg/mL of LP1 respectively) as compared to control (Figure 4(C1)); * *p* < 0.05). Similarly, a decrease in the expression level of MMP-9 was also observed (81.9%, 60.8% and 36.13% for 7.5, 15 and 30 µg/mL of LP1 respectively) as compared to control (Figure 4(C2)); * *p* < 0.05).

### 2.5. LP1 Induced Apoptosis by Inhibition of PI3K/Akt/mTOR Pathway

**Figure 5:**
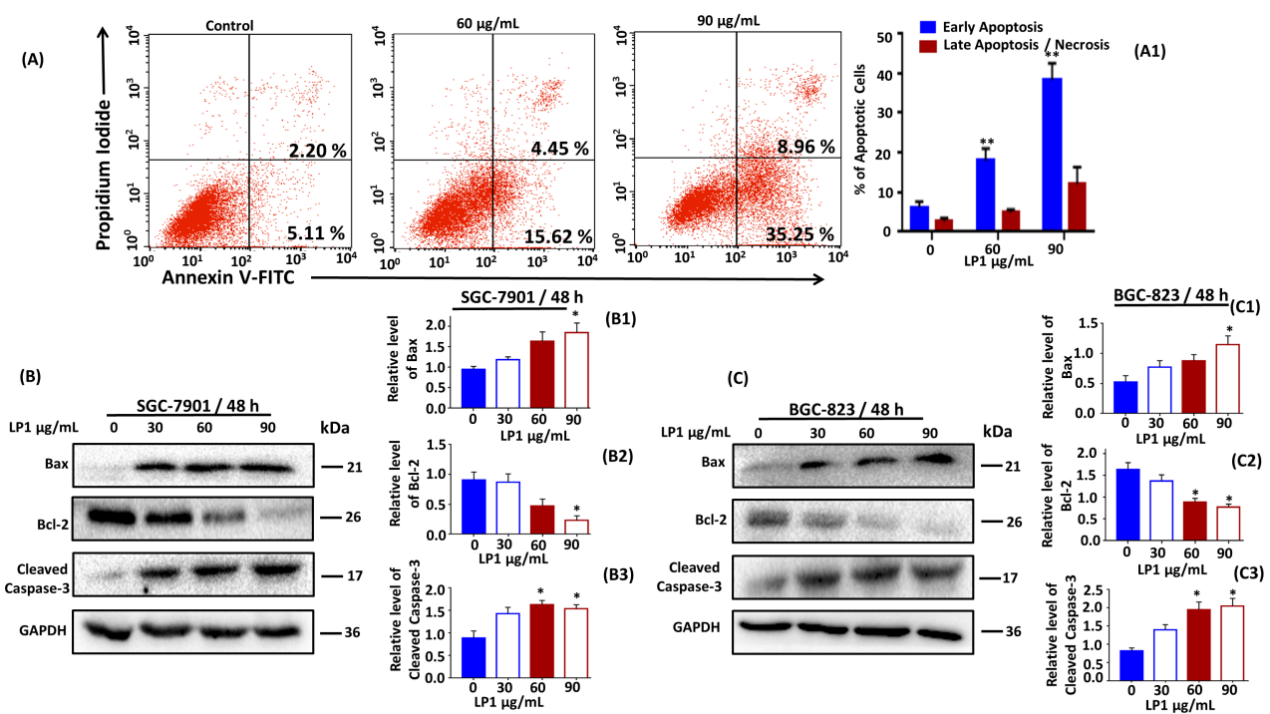
Induction of apoptosis and inhibition of PI3K/Akt/mTOR by LP1 in SGC-7901 cells. (**A**) SGC-7901 cells were treated with (0, 60, 90 µg/mL) and without LP1 for 48 h to analyze apoptosis by Annexin-V/PI staining. Numbers within the quadrant represent the percentage of cells (Annexin V+/PI−, lower right) and (Annexin V+/PI+, upper right). (**A1**) A number of apoptotic populations were counted. Results are expressed as means ± SD (** *p* < 0.01) of three independent experiments; (**B**) expression level of Bax, Bcl-2 and cleaved caspase-3 in SGC-7901 cells treated with various concentration (0, 30, 60, 90 µg/mL) of LP1 for 48 h were analyzed by western blot assay, GAPDH was used as an internal control. (**B1**–**B3**) Western blots were quantified by densitometry and results are expressed as means ± SD (* *p* < 0.05) of three independent experiments. (**C**) Expression level of Bax, Bcl-2 and cleaved caspase-3 in BGC-823 cells treated with various concentration (0, 30, 60, 90 µg/mL) of LP1 for 48 h were analyzed by western blot assay, GAPDH was used as an internal control. (**C1**–**C3**) Western blots were quantified by densitometry and results are expressed as means ± SD (* *p* < 0.05) of three independent experiments.

Flow cytometry was performed followed by Annexin V/Propidium Iodide staining, to examine the exposure of phosphatidylserine on the membrane surface which is an indication of apoptosis in cells. After treating the SGC-7901 cells with (0, 60 and 90 µg/mL) of LP1 for 48 h, the cells were exposed to flow cytometer to assess the percentage of apoptosis. The results indicated an increase in the percentage of apoptotic cells from 7.31% to 44.21% (Figure 5A and Figure 5(A1)); * *p* < 0.05, ** *p* < 0.01). Further, we performed western blotting assay to investigate the expression level of apoptosis-related key proteins (Bax, Bcl-2 and caspase-3) as shown in Figure 5B,C. For SGC-7901 cells, the expression level of Bax increased by 1.3, 1.7 and 2.0-fold (Figure 5(B1)); * *p* < 0.05); while the expression level of Bcl-2 decreased by 95.7%, 52.3% and 25.35% (Figure 5(B2)); * *p* < 0.05) dose dependently at 48 h, as compared to the control cells. The expression level of cleaved caspase-3 was increased by 1.6, 1.8 and 1.7-fold (Figure 5(B3)); * *p* < 0.05) as compared to the control cells, when treated with various concentration of LP1 at 48 h. For BGC-823 cells, the expression level of Bax increased by 1.5, 1.7 and 2.0-fold (Figure 5(C1)); * *p* < 0.05); while the expression level of Bcl-2 decreased by 94.0%, 60.5% and 50.05% (Figure 5(C2)); * *p* < 0.05) dose dependently at 48 h as compared to the control cells. 

The expression level of cleaved caspase-3 was increased by 1.4, 1.75 and 1.8-fold (Figure 5(C3)); * *p* < 0.05) as compared to the control cells, when treated with various concentration of LP1 at 48 h.

So, these results suggested that induction of apoptosis in SGC-7901 cells may be due to the intrinsic pathway. However, the further detailed investigation will confirm the role of LP1 in the induction of a precise apoptotic pathway.

**Figure 6:**
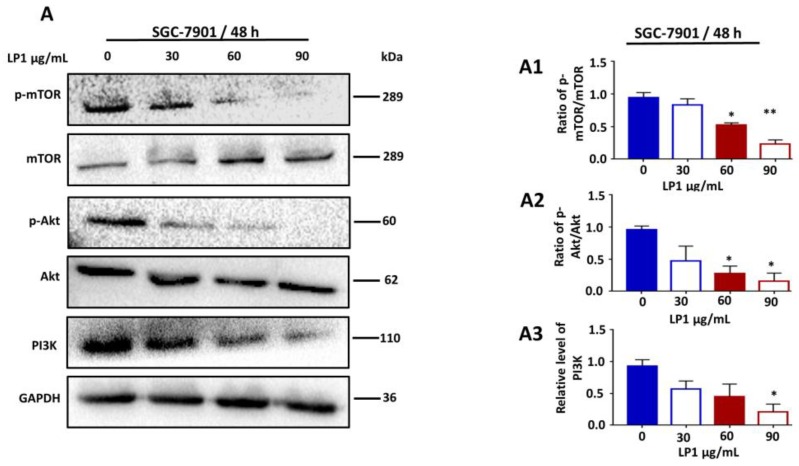
Inhibition of PI3K/Akt/mTOR pathway in SGC-7901 cells by LP1. (**A**) The expression levels of p-mTOR, mTOR, p-Akt, Akt and PI3K proteins were analyzed by western blotting assay. SGC-7901 cells were treated with various concentrations of LP1 (0, 30, 60, 90 µg/mL) for 48 h, GAPDH was used as an internal control. Data are expressed as means ± SD of three independent experiments. (**A1**–**A3**) Western blots were quantified by densitometry, results are expressed as mean ± SD and * *p* < 0.05, ** *p* < 0.01) of three independent experiments.

To explore Ser473 phosphorylation of Akt and Ser2448 phosphorylation of mTOR, we treated cells with different concentrations of LP1 for 48 h and results of western blotting showed decreased expression level of phosphorylated Akt and mTOR in SGC-7901 cells (Figure 6A). The ratio of p-mTOR/mTOR decreased by 88.29%, 55.39% and 24.80% (Figure 6(A1)); * *p* < 0.05, ** *p* < 0.01); the ratio of p-Akt/Akt declined by 50.03%, 29.14% and 16.74% (Figure 6(A2)); * *p* < 0.05); Similarly, the expression level of PI3K decreased by 40.11%, 31.32% and 19.15% (Figure 6(A3)); * *p* < 0.05) when treated with various concentration of LP1 for 48 h as compared to control cells. These results suggested that LP1 induced apoptosis by the inhibition of PI3K/Akt/mTOR pathway.

## 3. Discussion

Gastric cancer is the first leading cause of cancer-associated death in China and second in Asia, with only five years of survival rate after diagnosis [26,27]. So, there is a need for novel therapeutic natural agents with potential activity against cancer and having minimum side effects. LP1 was reported for its anticancer activity only in the lung cancer cell line A549 [19]. In this study, we evaluated the anticancer effect of LP1 on a number of cancer cell lines that is, SGC-7901, BGC-823, SKOV-3, HepG-2, MDA-MB-231, MCF-7 and normal cell lines GES-1 and HaCaT. The cancer cell lines are randomly chosen, based on the fact that these cell lines are derived from the cancers which are listed among the ten most fatal cancers around the world during 2018 [2]. Hence it is useful to study the effect of novel natural drugs on these cancers. Herein, we have found the gastric cancer cell lines (SGC-7901 and BGC-823) most sensitive to LP1 as compared to other cell lines (Figure 1). Moreover, we also found that the IC_50_ values for SGC-7901 and BGC-823 are almost similar (31.5 µg/mL and 40.7 µg/mL for SGC-7901 and BGC-823 cells respectively). In this study, we analyzed the distribution of cell population in different phases of the cell cycle after treating SGC-7901 cells with LP1. The results suggested that LP1 can arrest S phase of the cell cycle as the numbers of cells were significantly greater in this phase with respect to other phases of the cell cycle (Figure 2A and Figure 2(A1)). Although most studies found that autophagy arrests the M phase of the cell cycle, however, some investigations have been reported to show deviation from it. It is reported that when autophagy inducers (BH3 mimetic ABT 737, lithium, rapamycin, tunicamycin or nutrient depletion stereotypical) was added in different cell lines, they show heterogeneity in the cell cycle arrest. These drugs mainly arrested the G1 and S phase of the cell cycle [28]. Sarsaparilla (Smilax Glabra Rhizome) was reported to show in vitro and in vivo inhibitory effect on multiple cell lines by inducing autophagy, apoptosis and arresting S phase of the cell cycle [29]. Similarly, Cannabisin B was reported to induce autophagic cell death in HepG2 cells by inhibiting the Akt/mTOR pathway and blocking S phase of the cell cycle [30]. Hence autophagy-mediated S phase arrest of SGC-7901 cells is in harmony with the previously reported studies.

Autophagy is a type II cell death which involves in the isolation of cellular organelles, long-lived proteins and cytoplasmic parts; to form a double membrane structure called autophagosomes, these autophagosomes fuses with a lysosome and the modified structure is called autolysosome which is ultimately degraded [31]. Acridine orange staining indicated the formation of autophagic vacuoles called lysosomes in the acidic compartment of the cells by emitting orange light (Figure 3A,B). Furthermore, the results of electron microscope also revealed the formation of a large number of autophagosomes in the treated group (Figure 3C). PI3KCIII is involved in the activation and regulation of autophagy, it is an evolutionarily conserved protein and most reactive form of PI3K family and is associated with the regulation of enzymatic function, transfer of signals and membrane trafficking [32]. The confirmation of membrane trafficking by PI3KCIII was first time recognized after the identification of Vps34 (catalytic subunit) of PI3K and Vps15 (regulatory subunit) of PI4K in yeast [32,33,34,35]. Yeast genetic study reveals the presences of 34 autophagy-related genes, among them, 15 are essential for various autophagic pathways. There are two (E1 and E2) ubiquitin-like systems; E1 enzyme is similar to Atg7 and E2 is similar to Atg10. In this system, Atg12 is covalently conjugated with Atg5 [36,37]. The Atg12-Atg5 conjugation system plays a key role in the membrane elongation of autophagic vacuoles [38]. According to lately investigated reports, Atg12-Atg5 conjugation system directs the mitochondrial homeostasis and leads to cell death instead of nutrient-deprived or stress-induced autophagy [37]. Atg12-Atg5 conjugation system also functions like E3 to conjugate Atg8 (mammalian LC3) with PE (phosphatidylethanolamine), an amide bond is formed between the amino group of PE and the carboxyl-terminal of Atg8 which aid in the development of autophagosomes [39,40]. To understand whether LP1 induced autophagy is due to the activation of these autophagy-related proteins or not, we conducted western blotting assay and found 1.8-fold increase in the expression level of Atg7; 1.9-fold increase in the expression level of Atg5 and similarly, 1.7-fold increase in the expression level of Atg12 as compared to control. These proteins are associated with membrane elongation and accumulation of the spoiled proteins, cytoplasmic parts and intracellular organelles to form autophagosomes [23,24,25]. Induction of autophagy leads to the conversion of Atg8 from its soluble form (LC3 I) to an insoluble form (LC3 II) [41]. Western blotting results showed an increase in the conversion level of LC3I to LC3II as compared to control. These results provided a support for LP1 mediated induction of autophagy in SGC-7901 cells (Figure 3). The role of Beclin1 as autophagy stimulating and tumor-suppressing gene is well known since 1999. In the later years, it was confirmed that Beclin1 is mono-allelically deleted in human breast, brain, ovarian and prostate cancer [42,43,44,45]. The mutation was reported in Beclin1 binding protein UVRAG (UV irradiation Resistance-Associated Gene) which also regulate autophagy in human gastric cancer [46]. Moreover, the positive results of autophagy and inhibition of tumor in mice were monitored by transferring of the Beclin1 gene [42]. According to the reports, the role of Beclin1 as autophagy promoter is linked with the formation and activation of a complex between Vps34 and Atg14 which plays a crucial role in the formation of autophagosomes [32,47]. Our results showed an increase in the expression level of Beclin1 and Atg14 when treated with LP1 which further supports the induction of autophagy (Figure 3(D1,D5)).

Metastasis is a key obstacle in the diagnosis and treatment of cancer as it results in shortening of survival duration in patients [48]. According to reports, undue activation of Akt causes metastasis elevation in human gastric cancer [49]. However, Akt down-regulation inhibits tumor growth by decreasing migration and invasion [50]. Matrix metalloproteinases (MMPs) are zinc associated endopeptidases which play important role in tissue transformation. There are 28 different members in the MMPs family, among them MMP-2 and MMP-9 acts in degrading the type IV collagen that is a component of basement membrane. The degradation of basement membrane leads to the progression of metastasis in a variety of tumors [51,52]. In this study, we found that LP1 significantly suppressed the expression level of MMP-2 from 96.6% to 31.0% and MMP-9 from 81.9 to 36.13% (Figure 4(C1,C2)) which in turn leads to significantly reduced rate of wound healing, migration and invasion in SGC-7901 cells.

Apoptosis is a type I programmed cell death, in this type of cell death members of Bcl-2 protein plays the main role in the inhibition of caspase proteins and apoptosis [53,54]. Many factors are involved in the activation of apoptosis; the main feature includes activation of pro-apoptotic proteins and inhibition of anti-apoptotic proteins [55]. In this study when we treated the SGC-7901 cells for 48 h, the expression level of Bax increased 1.3, 1.7 and 2.0-fold (Figure 5(B1)) while the expression level Bcl-2 decreased 95.7%, 52.3% and 25.35% (Figure 5(B2)) in a dose dependent-manner, along with increase in the expression level of cleaved caspase-3 1.6, 1.8 and 1.7-fold (Figure 5(B3)). These results suggested the occurrence of apoptotic cell death in SGC-7901 cells.

PI3K/Akt/mTOR pathway is essential for the regulation of growth, proliferation, cell cycle, metastasis, apoptosis and autophagy [56,57,58]. Over-activation of this pathway results in the abnormal growth of cells which in turn leads to the survival of cancer cells and considered as the main barrier in the diagnosis and chemotherapeutic treatment of various types of cancers including gastric cancer [59,60]. It has been reported that amplification and mutation of PI3K in gastric cancer are associated with the deregulation of the PI3K/Akt/mTOR pathway [61,62,63,64]. In this study, we found that LP1 treatment down-regulated the PI3K/Akt/mTOR activation and suppressed the phosphorylation of Akt/mTOR (Figure 6A) in SGC-7901 cells. Thus these observations indicated that LP1 induced apoptosis that is due to the inhibition of the PI3K/Akt/mTOR pathway. 

We also confirmed the autophagy vacuolization in BGC-823, by staining the cells with acridine orange dye and found a large number of autophagic vacuoles in the LP1 treated cells. Further we investigated if the phenomenon of apoptosis exists in LP1 treated BGC-823 cells or not. In LP1 treated BGC-823 cells, an increase in the expression level of Bax, a decrease in the expression level of Bcl-2 and cleavage of caspase-3 was observed, indicating that cell death is due to autophagy and apoptosis. Since autophagy and apoptotic cell death is evidenced in two different types of aforementioned gastric cancer cells; hence we proposed that induction of autophagy and apoptosis in LP1 treated gastric cancer cells is independent of cell type. However, these are only basic findings; therefore, a further detailed study on other different cells is required to clearly reveal this fact.

Therefore, on the basis of these results, we found that LP1 induced autophagy at 24 h, which in turn leads to the induction of apoptosis by inhibiting PI3K/Akt/mTOR pathway at 48 h. It arrested the S phase of the cell cycle with the lower doses and inhibited invasion and migration. However, these are very preliminary investigation of LP1 on the gastric cancer cell line SGC-7901. There is a need for further investigation; so, our future studies will focus on the shortfalls of this study that is, assessing the correlation between LP1 induced autophagy and apoptosis (both in vivo/in vitro). Although the cytotoxic dose of LP1 used for this cell line is high and did not correlate to humans. In addition to other factors, we will also focus to find a number of strategies to investigate the maximum tolerated dose by steady-state plasma level concentration and perform PK/PD experiments on animal models. Our future targets will also include investigation of protein binding ability, half-life and drug delivery modes (endosomolytic agents, stimuli-responsive nanocarriers) of LP1 for both in vitro and in vivo study.

## 4. Materials and Methods

### 4.1. Reagents and Cell Culture

Atg7, Atg5, Atg12, Akt, Bax, Bcl-2, Beclin1, mTOR, MMP-9, MMP-2 and RIPA buffer were purchased from Proteintech (Wuhan, China). Atg14, LC3A/B, p-Akt (Ser473) and p-mTOR (Ser2448) were purchased from Cell Signaling Technology (Beverly, MA, USA). CCK-8 and Annexin-V/PI Kit were purchased from keyGEN BioTECH (Nanjing, China). Matrigel matrix was acquired from Corning Inc. (Corning, NY, USA) and acridine orange was obtained from Solarbio Science & Technology China. Human gastric cancer cell line SGC-7901, human ovarian cancer cell line SKOV-3, hepatoblastoma cells HepG2, human breast cancer cell lines (MDA-MB-231 and MCF-7), human keratinocytes HaCaT and normal epithelial gastric cancer cell line GES-1 were purchased from Shanghai cell bank, Chinese Academy of Sciences (Shanghai, China), while human gastric cancer cell line BGC-823 was provided as a gift from Bo Song from pathology and forensics department of Dalian Medical University. SGC-7901, BGC-823 and HaCaT cells were maintained in RPMI-1640 medium (HyClone Laboratories; Logan, UT, USA); SKOV-3, HepG2, MDA-MB-231, MCF-7 and GES-1 cell lines were maintained in Dulbecco’s modified Eagle’s medium (DMEM) (Hyclone; GE Healthcare Life Sciences, Logan, UT, USA), containing FBS (fetal bovine serum) 10% (Tianjin Haoyang Biological Products Technology Co., Ltd., Tianjin, China), penicillin (100 units/mL) and streptomycin (100 µg/mL) at 37 °C, in a humidified atmosphere with 5% CO_2_.

### 4.2. Cell Viability and Proliferation

All cell lines (1 × 10^5^) were cultured in 96 well plate, containing 200 µL RPMI-1640 and DMEMper well. Cells were allowed to grow overnight at 37 °C in a 5% CO_2_ atmosphere until confluency elevated from 70 to 80%. After washing with PBS, the cells were treated with different concentration of LP1 (0, 7.5, 15, 30, 60, 90 and 120 µg/mL). The cell medium was replaced with fresh RPMI-1640/DMEM (10% FBS) and CCK-8 (10 µL/well) was added after 24 h/48 h followed by incubation for 4 h. ELISA microplate reader was employed to determine the cell viability by monitoring optical density (O.D) at 450 nm. A similar procedure was adopted for cell proliferation assay; with different concentration of LP1 (0, 7.5, 17 and 30 µg/mL), after 0, 24, 48 and 72 h the number of the cell were counted by ELISA microplate reader (Biotek, Winooski, VT, USA) at O.D. 450 nm.

### 4.3. Phase Contrast Microscopy

SGC-7901, BGC-823 and GES-1 cells were seeded in 12 well plates for 24 h until 70 to 80% confluency was achieved. The cells were washed twice with PBS, treated with LP1 (0 and 90 µg/mL) and incubated for 48 h (37 °C, 5% CO_2_**,** humidified atmosphere). The cells were examined and photographed under phase contrast microscope.

### 4.4. Cell Cycle Analysis

SGC-7901 cells were treated with various concentrations (0, 7.5, 15 and 30 µg/mL) of LP1 for 48 h. The cells were trypsinized (without EDTA), washed twice with ice-cold PBS, centrifuged (2000 rpm/5 min, 4 °C), fixed in 70% ethanol and kept overnight. Next morning, the cells were collected by centrifugation (1200 rpm/4 min, 4 °C) and washed with ice-cold PBS. The cells (5 × 10^5^/mL) were treated with RNase (5 µg/mL) and incubated at 37 °C for 2 h. Propidium iodide (50 µg/mL) was added and the cells were incubated again at 37 °C for 30 min. The cell cycle was monitored with FACS-Calibur Cytometer (BD Biosciences, Heidelberg, Germany) within 60 min.

### 4.5. Electron Microscopy

After treating the SGC-7901 cells with and without LP1 for 24 h, the cells were trypsinized, washed twice with PBS, centrifuged (1500 rpm/5 min) and then the cell pellets of treated and control groups were fixed in 2.5% glutaraldehyde in 0.1 M phosphate buffer. Next, post-fixation was done in 1% osmium tetroxide for 2 h, washed twice with PBS and a series of dehydration was carried out by different concentration of ethanol ranging from 70% to 100%. After that, the samples were embedded in Epoxy resin and an ultramicrotome was used to cut them into thin sections. These thin sections were stained using saturated uranyl acetate and lead citrate. Finally, it was observed by JEM-1220 electron microscope (JEOL, Tokyo, Japan).

### 4.6. Acridine Orange Staining

SGC-7901 and BGC-823 cells were seeded in 12 well plates for 24 h until 70–80% confluency was achieved. The cells were washed twice with PBS, treated with different doses of LP1 (0, 30, 60 and 90 µg/mL) and incubated for 24 h (37 °C, 5% CO_2_**,** humidified atmosphere). The cells were trypsinized, centrifuged (1000 rpm/5 min) and treated with acridine orange according to the manufacturer’s instructions. The cells were kept in dark for 20 min at 37 °C, washed with PBS and transferred to the glass slide for inspection. A fluorescence microscope (*λ*_ex_ = 488 nm, *λ*_em_ = 515 nm) was employed to observe the cells with orange vacuole in five fields.

### 4.7. Cell Wound Healing Assay

SGC-7901 cells were seeded in 12 well plates and examined until reached to the 90% confluency. A sterile 200 µL pipette tip was used to scratch a wound on the cells and washed with PBS to remove debris. The wounded cells were treated with LP1 (0, 15 and 30 µg/mL) and photographed at 0 h, 24 h and 48 h under a phase contrast microscope. The wound healing rate was quantified by using ImageJ 1.50i (Wayne Rasband National Institute of Health, Bethesda, MD, USA) and calculated with the help of following formula (Aw − At)/A_w_ × 100. Where A_w_ is an area of the wound at 0 h (control) and A_t_ is the area of the wound at 24 h/48 h after scratching.

### 4.8. Cell Invasion and Migration Assay

Cell invasion assay was performed with Matrigel matrix and cell migration assay was performed without the mentioned gel matrix. Manufacturer’s instructions were followed to prepare Matrigel matrix. Briefly, after dilution, the Matrigel matrix was allowed to coagulate for 12 h (37 °C, 5% CO_2_, humidified atmosphere) in a transwell chamber (8 µm pore size; Corning Inc.). LP1 (0, 15 and 30 µg/mL) treated cells were trypsinized, centrifuged (1000 rpm/5 min) and seeded in a transwell (1 × 10^5^ cells/upper chamber). The upper chamber was filled with 200 µL RPMI-1640 medium (without FBS) and lower chamber was filled with 700 µL RPMI-1640 medium containing 10% FBS. The cells were permitted to invade and migrate for 24 h (37 °C, 5% CO_2_, humidified atmosphere). After that transwell chamber was detached from 24 well plate, the cells on the inner side (non-invasive/non-migrated) were removed with the aid of cotton swab and the cells on the lower side (invasive/migrated) were fixed in formaldehyde (4% in PBS) followed by staining with crystal violet (0.1% in ethanol, room temperature, 15 min). The PBS washed and room temperature dried chamber was subjected under a microscope (20× magnification) for image capturing. The numbers of cells on the lower sides of the transwell chamber were calculated in a double-blind manner by three different investigators.

### 4.9. Annexin V-FITC and Propidium Iodide Staining

SGC-7901 cells were treated with various concentrations (0, 60 and 90 µg/mL) of LP1 for 48 h (37 °C, 5% CO_2_, humidified atmosphere). The cells were trypsinized (without EDTA), washed twice with PBS, centrifuged (2000 rpm/5 min) and stained with Annexin V-FITC and PI according to the manufacturer’s instructions. The cells were kept in dark for 15 min, at 37 °C and FACS-Calibur Cytometer (BD Biosciences, Heidelberg, Germany) was employed to monitor the rate of apoptosis within 60 min.

### 4.10. Western Blotting

LP1 treated and untreated cells were lysed on ice for 30 min with radio immunoprecipitation assay (RIPA) buffer containing protease inhibitor cocktail and phosphatase inhibitor (Transgen Biotech, Beijing, China). After centrifugation (14,000 rpm/20 min, 4 °C), protein concentration was measured by using a BCA kit, lysate (20–40 µg) was subjected to gel electrophoresis and transferred to polyvinylidene fluoride membranes (Millipore, Billerica, MA, USA). The membrane was blocked with 5% fat-free milk in TBS-T (NaCl 150 mM, Tris-HCl 20 mM with pH 7.5 and Tween 20) for 1 h at room temperature. Following primary antibodies were diluted according to the manufacturer’s instructions and incubated over night at 4 °C with blocking buffer: Atg5 (catalog no. 10181-2-AP, 1:2000), Atg7 (catalog no. 10088-2-AP, 1:500), Atg12 (catalog no. 11122-1-AP, 1:2000), Beclin1 (catalog no. 11306-1-AP, 1:3000), Atg14 (number 96752, 1:1000), LC3A/B (number 4108, 1:1000), GAPDH (catalog no. 23931-1-AP, 1:500), Bax (catalog no. 23931-1-AP, 1:500), Bcl-2 (catalog no. 12789-1-AP, 1:1000), cleaved caspase-3 (catalog no. 25546-1-AP, 1:500), Akt (catalog no. 10176-2-AP, 1:1000), Phospho-Akt (Ser473,D9E, 1:2000), mTOR (catalog no. 20657-1-AP, 1:1000), Phospho mTOR (Ser2448, D9C2, 1:1000), PI3Kp110 (catalog no. 20584-1-AP, 1:1000), MMP-2 (catalog no. 10373-2-AP, 1:1000) and MMP-9 (catalog no. 10375-2-AP, 1:1000), Prior to treatment with secondary antibodies, goat anti-rabbit or goat anti-mouse isotype:IgG (catalog no. 23931-1-AP, SA00001-1, 1:500) HRP-conjugate; the membrane was washed three times with TBS-T and kept for 1 h at room temperature. An enhanced chemiluminescence detection kit was used to develop blots and ChemiDoc^TM^ XRS + Imager-Bio-Rad (Hercules, CA, USA) was employed to capture the images.

### 4.11. Statistical Analysis

Statistical analysis was performed by Student’s *t*-test and one-way analysis of variance (ANOVA) followed by Tukey’s Multiple Comparison Test at *p* < 0.05 using GraphPad Prism software. All the data are expressed as the mean ± standard deviation of values obtained from at least three independent experiments unless otherwise stated.

## 5. Conclusions

In conclusion, our study demonstrated that LP1 from *Lentinula edodes* C_91-3_ had in vitro anticancer potential against human gastric cancer cell line SGC-7901 and BGC 823. LP1 induced autophagy at 24 h which in turn leads to apoptosis at 48 h by the inhibition of the PI3K/Akt/mTOR signaling pathway. Autophagy and apoptosis were also observed in BGC-823 cells. Furthermore, LP1 arrested cell cycle at S phase and reduced the wound healing, invasion and migration rate by suppressing the expression level of MMP-2 and MMP-9. Thus these results suggested LP1 as a preliminary potential natural agent for gastric cancer treatment investigation. However, further detailed in vitro and in vivo investigations are required.
